# Solvent for Gall-Stones

**Published:** 1874-12

**Authors:** 


Dr. Jackson, of Beaver county, Pa., gives the following as the best solvent for biliary concretions, gall-stones, etc., he ever knew:

| | | |
|---|---|---|
| ℞.—Chloroform, | | |
| Ether, | aa | ℥ss |
| Ol. Terebinth, | | ℥j |
| Sacch. Alb., | | ℥ij |
| Mucil. Acac., | | ℥ij |

M. Sig.—Teaspoonful three times a day.

With this a milk and vegetable diet should be used; meats and fats shunned.

